# Medical Treatment at Isolation Hospitals

**Published:** 1900-11-17

**Authors:** 


### Medical Treatment at Isolation Hospitals.

There is much in modern sanitary science to bring one round again to the old idea that *le bon Dieu* dispenses pestilence and fever with good and kind intent, rather than that they are due to the machinations of the Evil One, and that one object at least which epidemics are intended to serve is to provide object lessons, by aid of which we may learn the deficiences in our sanitary administration. In sanitation, indeed, as in culinary affairs, the proof of the pudding is in the eating, and many communities discover, only when it comes to the proof, by way of epidemics, that the sanitary pudding provided by the health officials does not go down.

Just now it is Colchester which finds the fare provided not altogether to its liking, the experience furnished by an outbreak of diphtheria having led to complaints about the insufficiency of its isolation hospital, the delay which has occurred in the removal of cases, the undue haste with which some of the cases have been discharged, the lack of disinfecting apparatus, and other matters connected with the administration of the place. It is not for us to examine critically all the points which have been raised, although we may say that there seems plenty to support the demand made by some of the residents for the institution of a Local Government Board inquiry. Certain charges, however, which have been formulated in regard to the treatment of the cases admitted to the hospital are of general interest, and deserve to be considered quite apart from their bearing upon the management of the Colchester Isolation Hospital.

The point in regard to treatment which has been specially complained of is the non-use of antitoxin, it being asserted that notwithstanding all that is known as to the efficacy of this drug, and as to the importance of its free administration early in the course of the disease, it is not being so used. The medical officer of health, however, who has charge of the cases, is understood to say that he would use antitoxin if he thought it necessary.

This brings us face to face with the question how far a community can properly control the treatment within its own hospital, and how far it is ever right or justifiable for any outside influence, whether that of the sanitary authority, of public opinion, or of outside medical men, to come between the doctor and his patient.

It seems to us sufficiently obvious that it would be in the highest degree improper for a medical man placed in a position of trust and of authority to allow himself to be driven by popular demands, from whatever source they come, to do anything but what he thinks the best for each individual case trusted to his care; and this being so, while we admit to the full that the refusal of a medical officer to make use of antitoxin in the treatment of diphtheria would be a good and sufficient reason for the managing committee of a fever hospital bringing his conduct under review, and carefully reconsidering the question of his reappointment, we cannot admit that any medical man or any parent, on sending a case into the hospital, has any right to demand any particular line of treatment. Whoever is entrusted with the case must bring proper knowledge of his profession to bear upon it, and must do his best—that is all; and we do not see how it is possible to introduce any modification of this broad rule without taking away from the medical officer in charge that responsibility which at present lies upon him. There is, however, another side to this problem. Let us once grasp the enormous responsibility thus thrown upon a man who is sole medical officer to an institution of any sort, and more especially to such an institution as an isolation hospital—many of the patients in which are removed to it under compulsory powers, and the work in which, from its very nature as a part of a system of isolation, is done under conditions far removed from the supervision if not the criticism of public opinion—and let us grasp also what an act of faith and confidence it is to allow oneself to be taken, or to allow one's dearest child to be taken, and shut up for long weeks in such an institution, and then one begins to see how important it is that each community should secure the very best of medical attendance for those who, often for the benefit of the public rather than for any advantage they themselves gain from the process, are shut up in these isolation hospitals When people once grasp the importance of this question. they will, we are sure, feel very doubtful whether the system which is so common at the present time, of making attendance at the isolation hospital a mere appendix to the other duties of the medical officer of health, is the best that could be devised.

Like the celebrated Topsy many of the details of sanitary administration in England have "growed," and they persist because they are already in existence rather than because they are especially reasonable, and among such details is that which often relegates to an officer, appointed for quite a different purpose, the medical treatment of the patients in our isolation hospitals. Every year the work of the properly-equipped medical officer of health is becoming more and more specialised; he is becoming a scientist; he is great on statistics and the tabulating of returns; he is a chemist or bacteriologist; he is an expert on water, drainage, and unhealthy areas; he is almost a lawyer in his knowledge of sanitary law; and the drift of tendencies is year by year to make him less and less of a practical physician. In many towns, indeed, he is debarred from private practice. And all this is as it should be. The tendency to specialism is in many ways good and not to be resisted. But this involves fresh arrangements as to the division of labour, and we cannot but think that the time has come when every isolation hospital should have its proper staff, and that the remuneration given should be such as to command the services of the best men in actual practice. We say nothing against the existing medical officers of health, but in proportion as their work becomes a speciality, standing apart from the ordinary work of practice, the very serious responsibility of treating those who are shut up for the benefit of the community in isolation hospitals should be given into the hands of a staff selected from among men whose whole time is passed in the treatment of the sick.

